# The early life of a leaf‐cutter ant colony constrains symbiont vertical transmission and favors horizontal transmission

**DOI:** 10.1002/ece3.7900

**Published:** 2021-08-05

**Authors:** Zachary I. Phillips, Luke Reding, Caroline E. Farrior

**Affiliations:** ^1^ Department of Integrative Biology University of Texas Austin Texas USA

**Keywords:** costs of generalism, myrmecophile, ontogeny, social immunity, vertical transmission, virulence

## Abstract

Colonial organisms host a large diversity of symbionts (collectively, parasites, mutualists, and commensals) that use vertical transmission (from parent colony to offspring colony) and/or horizontal transmission to disperse between host colonies. The early life of some colonies, characterized by the dispersal and establishment of solitary individuals, may constrain vertical transmission and favor horizontal transmission between large established colonies. We explore this possibility with the miniature cockroach *Attaphila fungicola*, a symbiont of leaf‐cutter ants and the mutualist fungal gardens they cultivate. The early life of a leaf‐cutter colony is characterized by the dispersal of a female alate (winged “queen”) carrying a fungal pellet, and the subsequent establishment of a foundress (workerless “queen”) raising her incipient fungal garden and colony. Roaches hitchhike on female alates during leaf‐cutter nuptial flights, which strongly suggests that roaches are vertically transmitted to foundresses and their incipient colonies; however, weak compatibility between roaches and incipient gardens may constrain roach vertical transmission. Reciprocally, opportunities for horizontal transmission between large established colonies with abundant fungal gardens may weaken selection against roach‐induced harm (virulence) of incipient gardens. We use a laboratory experiment, behavioral observations, field surveys, and a transmission model to estimate the effect roaches have on the survivorship of incipient gardens and the frequency of roach vertical transmission. Contrary to traditional assumptions, our results indicate that roaches harm incipient gardens and predominantly use horizontal transmission between established leaf‐cutter colonies. Ultimately, “costs of generalism” associated with infecting disparate stages of a host's lifecycle (e.g., incipient vs. established colonies) may constrain the vertical transmission of roaches and a broad range of symbionts.

## Graphical abstract

It may be costly for symbionts to infect the "newborn" solitary stages of colonial hosts, constraining vertical transmission and favoring horizontal transmission between larger established host colonies. We explore this possibility using a miniature symbiotic cockroach of leaf‐cutter ants and their fungal gardens. Our study demonstrates that accounting for host colony lifecycle heterogeneity is important for understanding symbiont transmission dynamics and evolution.

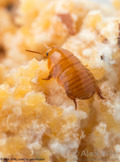

## 1 INTRODUCTION

Slowly something began to trickle into my brain: organisms are not just adults – they are lifecycles. – John Tyler Bonner, *Life Cycles: Reflections of an Evolutionary Biologist*

Colonial organisms represent important habitat patches of biodiversity, hosting diverse populations of parasites, mutualists, and commensals (herein, collectively referred to as “symbionts”). Although mature colonies can become enormous, colony lifecycles often commence with tiny solitary forms (Hölldobler & Wilson, 2009; Marti et al., 2015; Martin et al., 2018; Yang, 2007), and these humble beginnings can play a crucial role in the relationship between colonies and their symbionts. In particular, the early lives of colonies can influence patterns of between‐colony transmission, constraining vertical transmission and favoring horizontal transmission. For example, theory predicts that beneficial symbionts (zooxanthellae) of stony corals should be vertically transmitted from parent to offspring corals (Bennett & Moran, 2015; Bull et al., 1991; Hartmann et al., 2017, 2019; Herre et al., 1999); however, the same symbionts that benefit large established corals can harm their dispersing larvae (Hartmann et al., 2017, 2019). For some corals, these larva‐specific costs of infection favor coral acquisition of symbionts from the environment (horizontal acquisition) rather than from parents (vertical transmission), deviating from predictions that do not account for coral lifecycle heterogeneity (Hartmann et al., 2017, 2019).

For symbionts of eusocial insects, the solitary early life of a colony may present similar challenges to vertical transmission (herein, transmission from parent colony to incipient daughter colony), and accounting for colony lifecycle heterogeneity could dramatically alter predictions of transmission dynamics. Many colonies of ants, bees, wasps, and termites begin with just one or a few individuals and expand into colonies of thousands or millions of members, with resources growing in kind from meager to abundant (Hölldobler & Wilson, 2009; Tschinkel, 2011; Wheeler, 1910; Wilson, 1985). Leaf‐cutter ants in the genus *Atta* exemplify this transformation. They begin with one or a few gynes (female reproductives) each carrying a pellet of mutualist fungus and develop into complex insect societies maintaining abundant fungal gardens (Forti et al., 2017; Marti et al., 2015). Notably, the gyne is the only ant that passes through the entire colony lifecycle, including the solitary incipient stage. She changes in form and function from a winged gyne dispersing from her parent colony on a nuptial flight (*female alate*) into a wingless, workerless gyne raising an incipient colony (*foundress*), and finally, after the eclosure of her first brood of workers, she becomes the *queen* of the colony's ergonomic and mature stages (i.e., growth and reproductive stages, respectively)(Fernández‐Marín & Wcislo, 2005; Marti et al., 2015; Wilson, 1985).

At the scale of the colony, changes in form and function result from the codependent processes of fungal garden cultivation (Mueller et al., 2017) and sociogenesis, “the process by which colony members undergo changes in caste, behavior, and physical location incident to colonial development,” (Wilson, 1985, pp. 1489) creating a dynamic within‐nest environment for symbionts. During the lifecycle of a colony, these “guests” can experience changes in their host colony's size, resources, defenses, nest architecture, interaction networks, and other qualities that affect symbiont fitness (Cremer & Sixt, 2009; Hughes et al., 2008; Parmentier, 2020; Rynkiewicz et al., 2015; Tschinkel, 1993; Woodard et al., 2013). As such, colony stage‐dependent variation, including traits specific to the early life of a colony (Moreira et al., 2019), should be consequential for symbiont ecology and evolution.

We explore how the early life of a leaf‐cutter colony affects the between‐colony transmission of *Attaphila*, symbiotic cockroaches that exploit the ants and their mutualist fungal gardens (Bohn et al., 2021; Bolivar, 1901; Brossut, 1976; Djernæs et al., 2020; Nehring et al., 2016; Rodríguez et al., 2013; Waller & Moser, 1990; Wheeler, 1900). In Texas and Louisiana, *Attaphila fungicola* Wheeler is ostensibly common in the established colonies (i.e., ergonomic and mature) of its only available host, the Texas leaf‐cutter ant (*Atta texana* Buckley) (Moser, 1964, 1967a, 2006; Nehring et al., 2016; Phillips et al., 2017; Waller & Moser, 1990). Moser reports that “the roach inhabits the fungus gardens of most nests,” but does not provide a specific estimate of prevalence (1964, pp. 1048). At our field sites in Austin, TX, mature leaf‐cutter colonies with chronic roach infections (>5 years) survive and reproduce apparently unimpaired, so we use the neutral term “symbiont” instead of “parasite” to describe them (Phillips, 2021); however, the effect of roaches on incipient colony survival is unknown.

**FIGURE 1:**
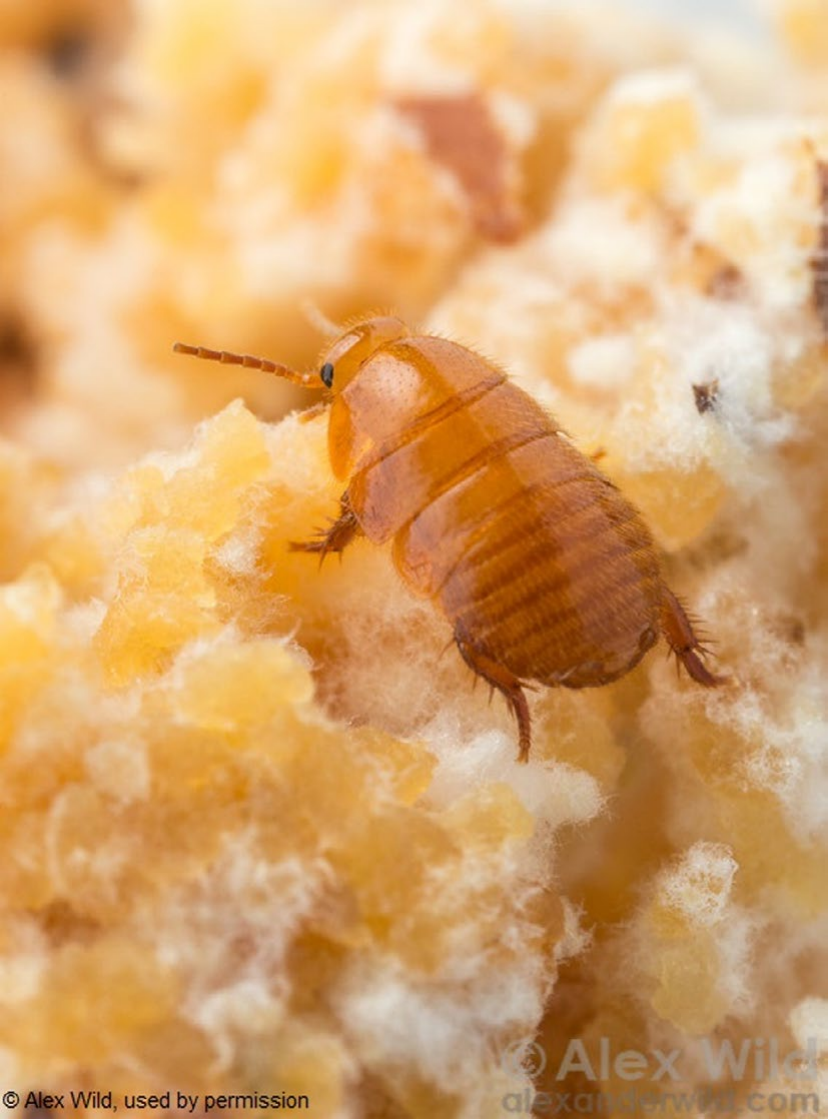
An adult female *Attaphila fungicola* roach on part of an established leaf‐cutter fungal garden

During the mass upheaval of a colony nuptial flight, roaches hitchhike on a small proportion of their host colony's dispersing female alates (<7%), and typically each “infected” female alate bears a single phoretic female roach (Moser, 1967a; Phillips, 2021; Phillips et al., 2017; Waller & Moser, 1990). Hitchhiking (i.e., *co‐dispersal*) on female alates has traditionally been interpreted as a behavior that initiates vertical transmission, likely because it suggests roaches remain with female alates as they become foundresses, and that roaches then *co‐establish* with foundresses and their incipient colonies (Djernæs et al., 2020; Moser, 1967a, 1967b); however, there is no evidence that roaches persist as infections through the incipient stage of colony development. Furthermore, recent findings indicate that hitchhiking roaches can abandon female alates after nuptial flights and subsequently ride leaves carried by foragers into the nests of established colonies (Phillips, 2021). Accordingly, hitchhiking on female alates may facilitate a complex mode of horizontal transmission between established colonies (“female alate‐vectored transmission,” Phillips, 2021) rather than vertical transmission to incipient colonies. In other words, roach *co‐dispersal* with female alates can be uncoupled from roach *co‐establishment* with foundresses and incipient colonies, and it remains unclear how frequently co‐establishment and thus vertical transmission occurs (Figure 1).

The low host quality of incipient colonies (extremely high mortality, low tolerance for disturbance, meager incipient gardens) may limit roach co‐establishment and constrain vertical transmission, favoring routes of horizontal transmission that bypass incipient colonies (direct or female alate‐vectored transmission between established colonies) (Moser, 1964; Phillips, 2021). Reciprocally, if roaches rarely or never use routes of vertical transmission that pass through incipient colonies, selection on roaches to avoid overexploiting and damaging incipient gardens should be weak (*weak incipient garden compatibility*). Alternatively, if roaches rely heavily on vertical transmission for dispersing between nests, they should be under strong selection to successfully co‐establish with incipient colonies, and to minimize harm and possibly provide benefits to incipient gardens (*strong incipient garden compatibility*) (Combes, 2001; Genkai‐Kato & Yamamura, 1999; Iritani et al., 2019; Lipsitch et al., 1996). To test whether roaches exhibit strong or weak incipient garden compatibility, and whether roaches primarily use vertical or horizontal transmission, we use a laboratory experiment to estimate the effect individual roaches have on the survivorship of low‐volume fungal gardens in artificial foundress chambers, and we use field surveys and a between‐colony transmission model to estimate the contribution of vertical transmission to roach prevalence among mature leaf‐cutter colonies.

## 2 METHODS

### 2.1 Field survey and collection

*Attaphila fungicola* female roaches and *A. texana* female alates were collected during nuptial flight preparations of mature leaf‐cutter colonies in May 2018 at Brackenridge Field Laboratory, Austin, TX (30.2840°N, 97.7780°W) (May 5, 21) and Hornsby Bend, Austin, TX (30.2327°N, 97.6374°W)(May 16). As thousands of alates and many thousands of nestmates gathered on nest mounds early in the morning, researchers ventured onto the mounds to collect alates and hitchhiking roaches. Eight out of 11 sampled mature *A. texana* colonies were infected with roaches (73% mature colony “infection” prevalence: 3/5 mature colonies with roaches at BFL, 5/6 mature colonies with roaches at Hornsby Bend). In total, 420 roaches were collected from 7,791 female alates (an average of 5.5% of female alates from colonies with roaches had a single roach attached). Roach prevalence per infected colony ranged from 2.2% to 6.8% of female alates bearing a single hitchhiking roach. Data have been deposited at the Dryad Data Repository (Phillips et al., 2021; https://doi.org/10.5061/dryad.8sf7m0cnt). Specimens of *A. fungicola* collected and not lost or destroyed during these and other experiments are accessioned (Accession number: UTIC255785) at the Insect Collection of University of Texas at Austin (https://biodiversity.utexas.edu/collections).

### 2.2 Incipient garden survivorship experiment

We collected paired female alate ants and roaches for use in the experiment, where alates collected from the field already had attached *A. fungicola* roaches. Using naturally paired ants and roaches ensured that both species came from the same natal colony, and thus controlled for potential intercolony differences (e.g., chemical profiles). We removed the wings of the female alates and placed the de‐winged alate (herein, “foundress”) and her attached roach in a 5 cm diameter container (“foundress chamber”) with 20 mg of fungal garden (“incipient garden”) from a laboratory colony. Notably, 20 mg is larger than the inoculum of fungus that foundresses initially regurgitate when founding a new colony under natural conditions (Marti et al., 2015). Long‐term survival in the laboratory of foundresses provided only with their inoculum is extremely rare, complicating experimentation and highlighting the extreme fragility of incipient colonies.

We compared the survivorship of incipient gardens and foundresses in two treatments: (a) foundress with roach treatment (i.e., foundress “infected”) and (b) foundress without roach treatment (i.e., foundress “uninfected”). We conducted the experiment after two nuptial flights from Brackenridge Field Laboratory (Flight 1: *n* = 53, Flight 2: *n* = 43, total *n* = 96 roach‐foundress pairings). Experiments were conducted under laboratory conditions described in Phillips et al. (2017), with all replicates kept at room temperature (22–24°C). Chambers were checked for 1 min every 24 hr in low‐light conditions to determine mortality of fungal gardens, foundresses, and roaches. The fungal garden was marked as effectively dead if it was dismantled and scattered in decaying clumps in upper and/or lower corners of the chamber and if the foundress did not tend any portion of the garden for at least 30 s (“uncaring” foundresses), or if the foundress was dead (without a caretaker, the fungal garden is effectively dead). Alternatively, the fungal garden was marked as living if the foundress tended a contiguous portion of the garden for at least 30s (i.e., the foundress’ head and mandibles maintained a position facing and over the garden, typically manipulating and antennating it: see end of Video S4, e.g., of foundress tending behavior). Additionally, 30 min following initial set‐up, each foundress chamber was observed for 3 min to determine if the inhabiting roach disturbed the incipient garden. Garden disturbance was scored if roach contact caused any observable movement, physical dislocation, or fragmentation of the garden, and subsequently, we categorized gardens as either “disturbed” (one or more observations of roach disturbance: see Videos [Link], [Link], [Link], e.g., of disturbance) or “undisturbed” (no observations of roach disturbance).

To test the effect of *A. fungicola* on incipient garden mortality, we used a mixed‐model Cox proportional hazards model with right‐censored daily mortality as the response variable and treatment (presence or absence of *A. fungicola*) as the explanatory variable. Nest location was nested within flight date as a random effect to account for variation in survivorship between flight dates and nests. Survival analyses were run using version 2.2 of the coxme package and the survival R package (Therneau, 2015). Additionally, we used a chi‐square test to determine whether there was a difference in the proportion of dead foundresses between the roach*‐*present and roach‐absent treatments. All analyses used R version 4.0.1 (R Core Team, 2020).

### 2.3 Between‐colony transmission model

**FIGURE 2:**
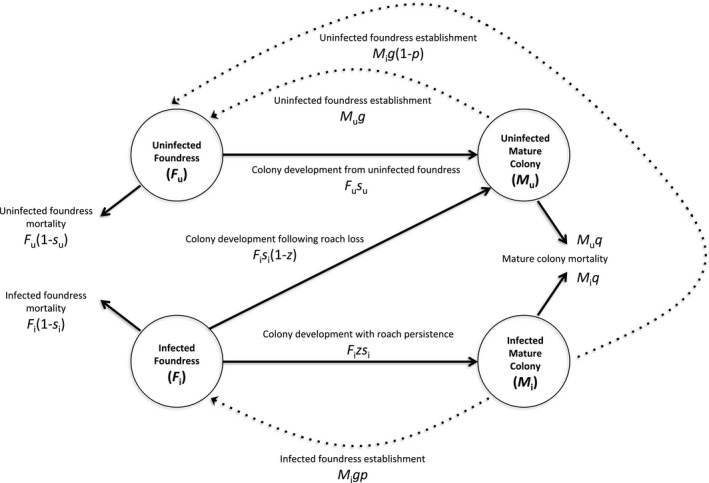
Diagram of model. See Table 1 for definitions of symbols

To explore the maximum contribution of vertical transmission to roach infection prevalence among mature colonies, we develop a simple model that assumes exclusive vertical transmission. We use this model to estimate mature colony infection prevalence (*V*) from foundress infection prevalence (*J*) and the effect of roach presence on the likelihood of a foundress reaching the mature colony stage (*δ*). See Figure 2 for a diagram of the model, and Table 1 for parameter symbols and definitions.

**TABLE 1:** Model symbols and definitions

Model symbols and definitions (with units)	
*F*_u_	Number of foundresses (incipient colonies) without roaches (individual)
*F*_i_	Number of foundresses (incipient colonies) with roaches (individual)
*M*_u_	Number of mature colonies without roaches (colony)
*M*_i_	Number of mature colonies with roaches (colony)
*s*_u_	Yearly proportion of foundresses without roaches (*F*_u_) reaching mature colony stage (colony/ind/year)
*s*_i_	Yearly proportion of foundresses with roaches (*F*_i_) reaching mature colony stage (colony/ind/year)
*δ* = *s*_i_/*s*_u_	Roach effect on the likelihood of foundress reaching the mature colony stage (unitless)
*z*	The persistence of inherited roaches across colony development, from foundress host to mature colony host (unitless)
*q*	Mortality rate of mature colonies (1/year)
*V*	Mature colony infection prevalence (i.e., proportion of mature colonies with roaches); *M*_i_/(*M*_i_ + *M*_u_) (unitless)
*J*	Foundress infection prevalence (i.e., proportion of foundress chambers occupied by roaches); *F*_i_/(*F*_i_ + *F*_u_) (unitless)

Parameter *δ* is the net effect of roach presence on foundress and incipient colony survivorship. A value of *δ* < 1 indicates that the roach is harmful to foundresses and their incipient colonies, *δ* = 1 that the roach is neutral, and *δ* > 1 that the roach is beneficial. To estimate *δ* from our incipient garden survivorship experiment, we use the inverse of a hazard ratio calculated from our survivorship analysis. This is a dimensionless measure of the effect roaches have on incipient garden survivorship.

Our field estimate of foundress infection prevalence, *J*, is not directly based on foundress infection prevalence (i.e., co‐establishing roaches) because roaches have not been observed in incipient colonies in our study region (Phillips et al., 2017) and we are not aware of estimates of foundress infection prevalence in any other region and for any other *Attaphila* species. This makes our best direct estimate of *J* zero and would indicate that vertical transmission does not occur. To account for the possibility that we have not observed these rare events, including the possible deposition of roach ootheca (egg cases) on alates or in foundress chambers, we estimate the maximum potential value of *J* from the maximum proportion of female alates with hitchhiking roaches collected from a single mature *A. texana* colony. This is likely a highly conservative estimate of *J* given that roaches co‐establishing with foundresses seem much rarer than roaches co‐dispersing with female alates (see Section 4). The maximum prevalence of roaches on a single nest mound in Austin, TX that has been recorded is 0.07 (On 15 May 2016, 50/719 female alates collected from a single nest mound surface preparing for nuptial flights at Brackenridge Field Laboratory). As we describe in “Model Results” below, using this conservative estimate of *J* = 0.07 helps estimate a conservative maximum possible contribution of roach vertical transmission to mature colony infection prevalence (*V*).

### 2.4 Model description

Our model is composed of four classes of ants: (a) foundress with roach (i.e., “infected” foundress) (*F*_i_); (b) foundress without roach (i.e., “uninfected” foundress) (*F*_u_); (c) mature colony with roach (i.e., “infected” mature colony) (*M*_i_); (d) mature colony without roach (i.e., “uninfected” mature colony) (*M*_u_). (Note, “infection/infected” here refers to the presence of a roach and/or its progeny in a host colony, not to microbial infections).

Mature colonies with roaches (*M*_i_) are generated in our model by the development of foundresses with roaches (*F*_i_) that survive to colony maturity with their roach infection intact. This is determined by the rate that foundresses with roaches reach the mature colony stage (*s*_i_) and the persistence of inherited roaches through colony development (*z*). If we assume mature colonies with roaches die at rate *q*, the change of mature colonies with roaches over time is as follows:

(1) $$\frac{dM_{i}}{\mathrm{dt}}=F_{i}s_{i}z‐M_{i}q$$

Mature colonies without roaches (*M*_u_) are generated by foundresses without roaches that survive to colony maturity (*F*_u_), determined by the rate that foundresses without roaches reach the mature colony stage (*s*_u_), and by the rate that foundresses with roaches (*F*_i_) lose their inherited roaches and reach the mature colony stage (*s*_i_(1 − *z*)).

(2) $$\frac{dM_{u}}{\mathrm{dt}}=F_{u}s_{u}+F_{i}s_{i}\left(1‐z\right)‐M_{u}q$$

**FIGURE 3:**
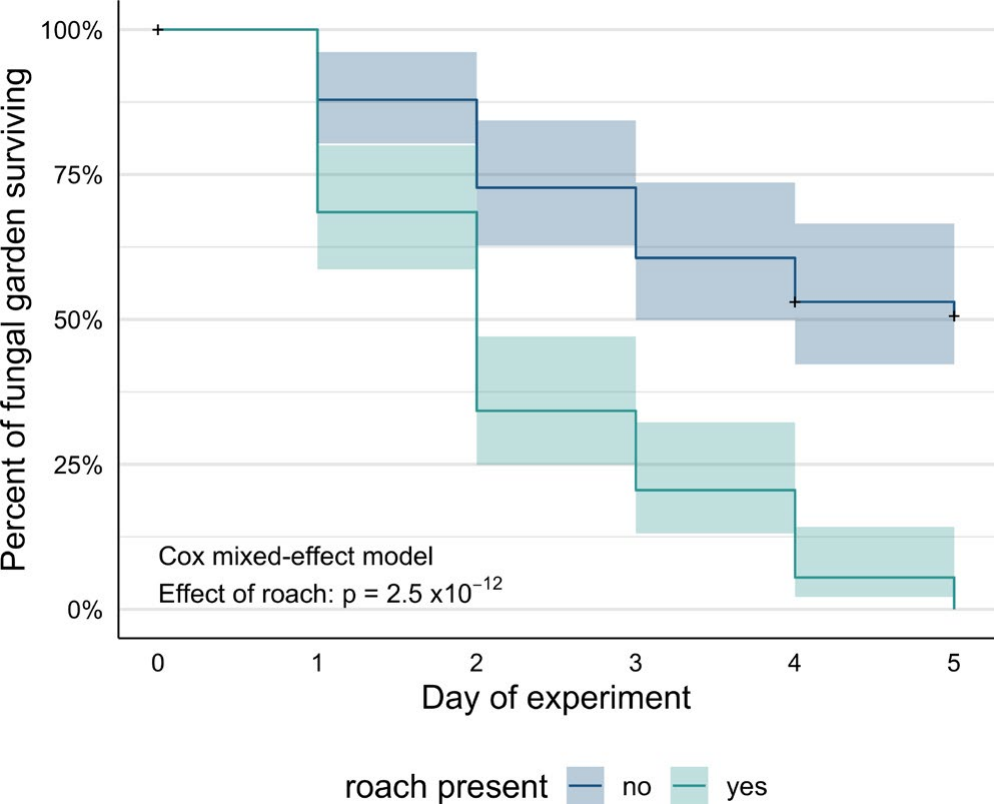
Survivorship of incipient fungal gardens in the presence or absence of *Attaphila* cockroaches (1 roach per garden). This comparison indicates that an accelerated rate of garden failure is associated with presence of a roach (see Section 4 for possible causes of this effect)

We assume that foundresses with roaches that lose them during colony development (e.g., the roaches die) are as likely to reach colony maturity as foundresses that maintain roaches through colony development (*s*_i_). In other words, we assume that the likelihood of foundresses reaching the mature colony stage is independent of the duration of roach infections. This assumption is consistent with our experimental results, which indicate *A. fungicola* has a rapid effect on low‐volume fungal garden survivorship (Figure 3). We also assume that mature colonies with roaches die at the same rate as mature colonies without roaches (*q*). This assumption is based on observations of similar nest surface frequencies of *A. fungicola* (i.e., similar proportions of female alates with hitchhiking roaches during nuptial flight preparations of a given infected nest: roughly 2%–7%) over a span of 5 years with no apparent reduction in colony health or size of nuptial flights. This assumption is also consistent with the general prediction that symbionts of large, long‐lived colonies are likely to evolve relatively low virulence (Hughes et al., 2008).

Given these assumptions, the mature colony infection prevalence (*V*), foundress infection prevalence (*J*), and the effect of *A. fungicola* on the likelihood of a foundress reaching colony maturity (*δ*) are defined as the following, respectively:

(3) $$V\equiv \frac{M_{i}}{M_{i}+M_{u}}$$

(4) $$J\equiv \frac{F_{i}}{F_{i}+F_{u}}$$

(5) $$\delta \equiv \frac{s_{i}}{s_{u}}$$

By solving Equations 1 and 2 at equilibrium, and using the above relationships (Equations (3), (4), (5)), we find *V* defined as a function of *δ*, *J,* and *z*.

(6) $$V=\frac{\delta z}{1/J‐1+\delta }$$

We use Eq. 6 to answer the following question: What is the maximum proportion of mature colonies that could be infected through the vertical transmission of roaches to foundresses (*V*_max_)? In other words, what is the maximum proportion of mature colonies that could acquire roaches that co‐disperse with female alates, then co‐establish with foundresses, and subsequently persist as colony infections until colony maturity? First, we estimate *V*_max_ based on our laboratory estimate of the roach effect on incipient fungal garden survivorship (*δ = *0.3, the inverse of hazard ratio 3.36, see Section 3). Second, we estimate *V*_max_ under the conservative assumption that roaches have no effect on incipient fungal garden survivorship (neutral, *δ* = 1). By “conservative assumption,” we specifically mean an assumption that selects parameter values deviating from more realistic values (i.e., values based on experiments, surveys, or natural history observations) in a way that maximizes model estimates of *V*.

For all estimates, we make the conservative assumption that roaches are never lost or cleared after occupying an incipient colony's foundress chamber (*z*_max_ = 1). In nature, *z* < 1 is certainly more accurate. Foundresses, for instance, have been observed attacking and killing roaches (Phillips et al., 2017). As discussed above, our estimate of *J* from female alate infection prevalence (*J* = 0.07) is likely a significant overestimate of foundress infection prevalence and therefore likely inflates our model estimates of *V*.

Note: We do not include the difference equations for infected foundresses (*F*_i_) and uninfected foundresses (*F*_u_) because they do not alter the result of Eq. 6 derived from the difference equations for infected and uninfected mature colonies (see Equations 1 and 2, respectively). The parameters *g* (*g* = number of foundresses produced/mature colony) and p (p = proportion of infected foundresses produced/mature colony) included in the model diagram also do not alter the result of Equation 6; we assume the production of female alates does not directly effect changes in the number of mature colonies. The model excludes male *A. fungicola* because they are generally absent during nuptial flights (Phillips et al., 2017; Waller & Moser, 1990). It has been proposed that *A. fungicola* are parthenogenetic in Louisiana (Waller & Moser, 1990) where no male *A. fungicola* have been collected from *A. texana* colonies.

## 3 RESULTS

### 3.1 Experiment results

*Attaphila fungicola* has a negative impact on the survivorship of incipient gardens when both “uncaring” and dead foundresses are included in the category of nonsurviving gardens (Figure 3; hazard ratio = 3.36, *z* = 7.01, *p* < 0.001). If only “uncaring” foundresses are included in the analysis, *A. fungicola* still has a negative impact on the survivorship of incipient gardens (hazard ratio = 4.44, *z* = 6.68, *p* < 0.001). The proportion of dead foundresses does not differ between treatments (chi‐squared test, *X* ^2^ = 0.659, *df* = 1, *p* = 0.417). Roaches were observed disturbing incipient gardens in 62.5% of foundress chambers (*n* = 96, 95% confidence interval: 52.0%–72.2%).

### 3.2 Model results

By incorporating our conservative values of *z* and *J* (*z* = 1, *J* = 0.07) and our laboratory estimate of *δ* (*δ* = 0.3) into Equation 6, we calculate that *V* = 0.02 (2% mature colony infection prevalence). Under these same conditions, but assuming roaches have no effect on the likelihood of a foundress reaching the mature colony stage (*δ* = 1), we calculate that *V* = 0.07 (7% mature colony infection prevalence). Our field survey estimate of mature colony infection prevalence is *V* = 0.73 (73% mature colony infection prevalence, see Section 2.1). A simple comparison of our model estimate of *V* based on our laboratory estimate of *δ* (*V* = 0.02) with our field survey estimate of *V* (0.73) indicates that strict vertical transmission could at most produce roughly 3% (0.02/0.73) of the proportion of infected mature colonies surveyed in the field. A similar comparison of our model estimate of *V* when we assume the roach has no effect on incipient garden survival (*V* = 0.07) with our field survey estimate of *V* (0.73) indicates that strict vertical transmission could at most produce about 10% (0.07/0.73) of the proportion of infected mature colonies surveyed in the field. Thus, both conservative model estimates of *V* indicate that vertical transmission is responsible for at most a small proportion (3% or 10%) of roach prevalence among surveyed mature colonies.

## 4 DISCUSSION

Symbionts inherited by host propagules (e.g., plant seeds, coral larvae, ant gynes) must *co‐disperse* and *co‐establish* with propagules for vertical transmission to be successful (Bibian et al., 2016). As a consequence, accounting for challenges that symbionts face during both host dispersal and establishment can help identify constraints on vertical transmission.

The propagules of leaf‐cutter ant colonies are gynes and the mutualist fungus they carry and care for, and the early life of a colony is marked by their dispersal (winged female alates carrying fungal pellets) and establishment (workerless foundresses raising incipient fungal gardens) (Helms, 2018; Marti et al., 2015; Moser, 1967a). During leaf‐cutter nuptial flights, the symbiotic cockroach *A. fungicola* hitchhikes on female alates (co‐dispersal), a behavior that strongly suggests roaches are vertically transmitted to incipient colonies (Moser, 1967a; Waller & Moser, 1990); however, roach co‐establishment with foundresses may be limited by weak compatibility with incipient gardens. The collective results of our experiment, behavioral observations, field surveys, and model indicate that roaches are weakly compatible with incipient gardens, that they at most rarely use vertical transmission, and that they primarily use horizontal transmission between established colonies.

Given the extreme fragility of incipient fungal gardens, we would expect selection for compatibility with incipient gardens to be strong for any vertically transmitted symbiont and for such symbionts to avoid harming or to even benefit incipient gardens during co‐establishment (Fries & Camazine, 2001; Genkai‐Kato & Yamamura, 1999; Herre et al., 1999; Lipsitch et al., 1996). In contrast, our results suggest roaches have evolved fixed responses to robust gardens rather than plastic behaviors that can be attuned to delicate gardens. In artificial foundress chambers, we observed roaches feeding on and rubbing against gardens (the latter may help the roaches acquire a colony's chemical profile)(Nehring et al., 2016) (see Videos [Link], [Link], [Link]), behaviors that are likely harmless to established gardens but could be catastrophic to incipient gardens and responsible for their accelerated failure (Figure 3). Also, roaches appeared to stress foundresses, consistent with observations from a previous study (Phillips et al., 2017). By antagonizing a foundress, a roach could indirectly cause significant damage to the garden (Moreira et al., 2019) (see Video S4, e.g., of a foundress turning away from her garden while grooming a roach off of her body; note, these interactions were observed but not scored as disturbances in our “incipient garden survivorship experiment” because they do not involve direct contact between roach and garden). Lastly, roaches might act as vectors of “hyperphoretic spores” and microbial garden diseases that kill incipient gardens (Di Prisco et al., 2011; Hughes et al., 2004; Moreira et al., 2019; Moser & Blomquist, 2011).

### 4.1 Mixed‐mode transmission between colonies

*Attaphila fungicola* vertical transmission may occur rarely, with populations of roaches using both vertical and horizontal transmission (i.e., mixed‐mode transmission)(Antonovics et al., 2017; Ebert, 2013). The only field observation that ostensibly describes roaches co‐establishing with incipient colonies notes *A. fungicola* in “new burrows made by [*A. texana*] queens” (Moser, 1967a, pp. 304). Other field observations suggest co‐establishment and vertical transmission are rare. Roaches have not been collected from *A. texana* foundress chambers in central Texas (Phillips et al., 2017), nor in incipient nests of its sister species *Atta mexicana* in Organ Pipe Cactus National Monument, AZ, and attempts to have these roaches co‐establish with foundresses have been unsuccessful (Phillips et al., 2017; pers. communication Alex Mintzer). Also, in northern Mexico, individuals of an unidentified species of *Attaphila* were observed running around independently of nearby *A. mexicana* foundresses searching for nest sites (Sánchez‐Peña, 2005) suggesting that these roaches had abandoned foundresses before vertical transmission could be completed (i.e., roach co‐dispersal uncoupled from co‐establishment).

If *Attaphila* roaches exhibit mixed‐mode transmission, perhaps encounters with foundress predators (e.g., armadillos, grackles, myrmecologists) influence a roach's decision to either take a chance remaining with a foundress likely to die (vertical transmission) or abandon the foundress and risk seeking an established colony (female alate‐vectored horizontal transmission**)**. In north Texas, a roach jumped off of a foundress seeking a nest site and disappeared into the grass, an escape apparently prompted by a researcher's collection of the foundress (U. G. Mueller, personal communication). Also, it is possible that roaches deposit ootheca (egg cases) on female alates or with foundresses before abandoning them; however, in field experiments where roaches were released while attached to female alates and foundresses, this was not observed (Phillips, 2021). In another study, roaches deposited ootheca within a few days of being collected with female alates during nuptial flight preparations, suggesting ootheca deposition would not have occurred during the nuptial flight itself (Waller & Moser, 1990). Even in the unlikely scenario that every hitchhiking roach attaches an ootheca to its co‐dispersing female alate, or deposits an ootheca during co‐establishment with a foundress, and assuming that ootheca and potentially accompanying adult roaches are harmless to incipient gardens, our model predicts vertical transmission would still occur infrequently (under these conservative conditions, our model predicts that the maximum proportion of mature colonies infected through vertical transmission is 10%; see Section 3.1).

Overall, the disparity between high mature colony prevalence and low female alate and foundress prevalence of roaches suggests vertical transmission is rare—unless roaches are somehow beneficial to incipient colonies and colonies with roaches disproportionately reach maturity. Our experiment indicates no such mutualism occurs (Figure 3). Infrequent vertical transmission could still play an important role in roach population dynamics and evolution, and vertical transmission might occur at higher frequencies in areas where the density of established leaf‐cutter colonies is low and there are fewer opportunities for horizontal transmission (e.g., range frontiers) (Mueller et al., 2011). Also, *Attaphila* individuals, “strains” and species could vary in their compatibility with incipient colonies, creating within‐species and between‐species variation in the frequency of vertical transmission. A comparative analysis of transmission strategies among *Attaphila* might reveal conditions that facilitate vertical transmission, but we know little about the life histories of most species or how many species exist (Bohn et al., 2021; Bolivar, 1901; Brossut, 1976; Djernæs et al., 2020; Nehring et al., 2016; Rodriguez et al., 2013; Sánchez‐Peña, 2005; Wheeler, 1900).

### 4.2 Potential roach strategies for mitigating virulence during co‐establishment

Virulence (i.e., symbiont‐induced harm to a host) can be adaptive or nonadaptive for symbionts (Bull, 1994; Leggett et al., 2013). A common model of adaptive virulence frames it as a property emerging from the trade‐off between transmission period and transmission rate: Increasing within‐host reproduction is costly because it increases virulence and reduces symbiont transmission period (i.e., kills the host faster), but beneficial because it increases symbiont transmission rate (i.e., rate of infection of new hosts) (Bull, 1994; Day, 2003). In contrast, the roach‐induced harm observed in our experiment probably represents nonadaptive virulence (Figure 3). A Texas leaf‐cutter foundress raises her incipient colony in a closed‐off (“claustral”) underground chamber (Marti et al., 2015), the same small space a vertically transmitted roach would presumably occupy during co‐establishment. If a roach contributes to the death of an incipient garden, it likely seals the fate of itself and its progeny in a shared grave with garden and foundress.

Nonadaptive virulence can be described as “virulence of no selective value per se…a coincidental byproduct of [symbiont] evolution in a different host species” (Bull, 1994, pp. 1424–1425). As this suggests, a major cause of nonadaptive virulence is infecting the “wrong” host, a host that a symbiont has not co‐evolved with and may not be compatible with (i.e., a host outside of the symbiont's host range) (Bull, 1994; Combes, 2001; Leggett et al., 2013). Although the “wrong” host often refers to an incompatible host species or strain, here we use it to refer to a potentially incompatible colony lifecycle stage, the incipient colony, which for *Attaphila* represents a radically different host environment than an established colony. Under this premise, roach behaviors that harm incipient colonies could arise as a byproduct of roach co‐evolution with established colonies. For example, if roaches have evolved an adaptive attraction to fungal gardens in the garden‐rich environment of established colonies, the same attraction may be nonadaptively virulent when expressed in the garden‐poor environment of incipient colonies. As discussed above, roaches might be able to mitigate this harm by adjusting their behavior during co‐establishment to avoid incipient gardens (i.e., behavioral plasticity, Leggett et al., 2013), or by exclusively using “behavior‐less” ootheca to co‐establish with incipient colonies (i.e., ontogenetic niche shift, ten Brink & de Roos, 2017; Werner & Gilliam, 1984).

Additionally, roaches may be able to mitigate harm by targeting leaf‐cutter co‐foundresses instead of solitary foundresses. Leaf‐cutter foundresses can join together to start a new colony, and these co‐foundress collectives exhibit higher survivorship and produce larger incipient gardens than solitary foundresses (Cahan & Julian, 1999; Mintzer, 1987). As a consequence, co‐foundresses and their incipient gardens could exhibit a greater tolerance for roaches (Ayres & Schneider, 2012; Cremer et al., 2018; Pull et al., 2013), increasing the likelihood of both roach and incipient colony survival; however, roaches have been observed abandoning co‐foundresses during the excavation of new colonies (Phillips, 2021; Phillips et al., 2021), suggesting that if roaches do infect incipient colonies, targeting co‐foundresses may not be a preferred strategy.

In general, abundant resources during co‐establishment should reduce the risk of symbiont overexploitation and catastrophic damage. Consider the early life of an ant‐plant‐homopteran mutualism, one in which a sap‐sucking scale insect (the homopteran) co‐establishes with an ant foundress on a myrmecophytic tree (Gaume et al., 1998). Although scale insects can be vectors of disease (Brown, 2016), and infestations can damage host plants (Golan et al., 2015), the sap‐sucking of one or a few scale insects during co‐establishment is unlikely to mortally wound a tree and doom the tripartite symbiosis. Now imagine if leaf‐cutter foundresses initiated colonies with tree‐sized fungal gardens instead of seed‐sized fungal gardens. Presumably a roach in this scenario would be innocuous during co‐establishment regardless of its behavior, and vertical transmission would not be constrained by incipient gardens.

### 4.3 Costs of generalism may constrain vertical transmission

Vertical transmission from parent to daughter incipient colonies requires both roach *encounters* with and *compatibility* with incipient colonies (Combes, 2001). Hitchhiking on female alates (co‐dispersal) facilitates encounters with incipient colonies because roaches simply have to remain with female alates as they transition into foundresses. Indeed, these easy encounters seem to be the basis for assuming hitchhiking is a first step in vertical transmission, and that co‐dispersal is tightly linked to co‐establishment; however, vertical transmission also requires compatibility with *both* incipient colonies and established colonies, while horizontal transmission requires compatibility with *only* established colonies. In the first case (vertical transmission), a roach must be a “generalist” of host colony lifecycle stages, while in the latter case (horizontal transmission), a roach can be a “specialist” of just established colonies. As a consequence, costs of generalism may ultimately constrain vertical transmission, not horizontal transmission, and attenuate the link between roach co‐dispersal and co‐establishment.

Some authors have divided costs of generalism that constrain symbiont compatibility with distinct hosts (i.e., restrict host range) into two categories: ecological costs and evolutionary costs (Benmayor et al., 2009; Leggett et al., 2013). Symbionts are susceptible to ecological costs when their potential hosts vary in quality, a scenario “analogous to that assumed in optimal foraging theory, where patches vary in quality” (Benmayor et al., 2009, pp. 764). In this context, ecological costs for symbionts are opportunity costs that result from infecting lower‐quality hosts instead of higher quality hosts (Bull, 2006; Heineman et al., 2008). Incipient leaf‐cutter colonies are intrinsically lower‐quality hosts than established colonies, possessing much higher mortality rates, fewer resources, and a lower tolerance for disturbance than established colonies. As such, we would expect vertical transmission from parent to daughter incipient colonies, but not horizontal transmission between established colonies, to impose strong ecological costs of generalism on roaches. Evolutionary costs of generalism associated with roach vertical transmission are less clear. Evolutionary costs arise if a symbiont's ability to infect one kind of host (the “novel host”) is associated with reduced performance in another kind of host (the “original host”)(Benmayor et al., 2009; Leggett et al., 2013). These costs could result from antagonistic pleiotropy between roach traits enhancing performance in incipient colonies (e.g., avoidance of fungal garden) and traits enhancing performance in established colonies (e.g., attraction to fungal garden).

Although many insect societies found colonies with just one or a few individuals (“independent founders”), some found colonies with a large number of individuals (“dependent founders”) (Cronin et al., 2013; LeBrun et al., 2013; Vargo & Porter, 1989). Army ants reproduce through a process called “colony budding,” in which a new queen accompanied by a large group of nestmates break off from their parent colony to form a new colony (Cronin et al., 2013). Budding allows an army ant colony to effectively skip the incipient stage and begin its life as an established colony (Cronin et al., 2013; Denny et al., 2004; Kronauer et al., 2010; Rettenmeyer et al., 2011). As a consequence, the vertical transmission of army ant symbionts entails transmission from one established colony (parent) to another (daughter) and should be less constrained by costs of generalism than the vertical transmission of leaf‐cutter symbionts such as *Attaphila*. Consistent with this possibility, albeit without invoking costs of generalism, Berghoff et al. (2009) and Łukasik et al. (2017) argue that army ant colonies should be more susceptible than independent founders (e.g., leaf‐cutters) to inheriting colony symbionts such as phoretic mites (Berghoff et al., 2009) and socially transmitted microbes (Łukasik et al., 2017).

If leaf‐cutter colonies were to reproduce through colony budding as army ants do, how would this affect *Attaphila* transmission? The vertical transmission of *Attaphila* might be less constrained by costs of generalism, and daughter colonies would likely inherit roaches more frequently.

## 5 CONCLUDING REMARKS

Colonies are lifecycles, and many begin with just one or a few individuals. From the perspective of a colony symbiont, the solitary early life of a colony represents a radically different host environment than that of a large established colony. Compared to established colonies, incipient colonies possess few resources, succumb easily to disturbance, and suffer high rates of mortality. All else being equal, incipient colonies are lower‐quality hosts than established colonies, and infecting the former instead of the latter can be costly. Across a broad range of host‐symbiont systems, these costs may constrain routes of vertical transmission that pass through incipient colonies and favor routes of horizontal transmission that bypass them.

## CONFLICT OF INTEREST

The authors have no conflicts of interests.

## AUTHOR CONTRIBUTIONS

**Zachary I. Phillips:** Conceptualization (lead); Data curation (equal); Formal analysis (equal); Funding acquisition (equal); Investigation (lead); Methodology (lead); Project administration (lead); Resources (lead); Software (supporting); Visualization (equal); Writing‐original draft (lead); Writing‐review & editing (lead). **Luke Reding:** Data curation (equal); Formal analysis (equal); Investigation (supporting); Methodology (supporting); Software (lead); Visualization (equal); Writing‐review & editing (supporting). **Caroline E. Farrior:** Conceptualization (supporting); Formal analysis (equal); Funding acquisition (equal); Methodology (supporting); Supervision (supporting); Visualization (equal); Writing‐original draft (supporting); Writing‐review & editing (supporting).

## Supporting information

Video S1

Video S2

Video S3

Video S4

## References

[ece37900-bib-0001] Antonovics, J., Wilson, A. J., Forbes, M. R., Hauffe, H. C., Kallio, E. R., Leggett, H. C., Longdon, B., Okamura, B., Sait, S. M., & Webster, J. P. (2017). The evolution of transmission mode. Philosophical Transactions of the Royal Society B: Biological Sciences, 372, 20160083. 10.1098/rstb.2016.0083 PMC5352810 28289251

[ece37900-bib-0002] Ayres, J. S., & Schneider, D. S. (2012). Tolerance of infections. Annual Review of Immunology, 30, 271–294. 10.1146/annurev-immunol-020711-075030 22224770

[ece37900-bib-0003] Benmayor, R., Hodgson, D. J., Perron, G. G., & Buckling, A. (2009). Host mixing and disease emergence. Current Biology, 19, 764–767. 10.1016/j.cub.2009.03.023 19375316 PMC7126095

[ece37900-bib-0004] Bennett, G. M., & Moran, N. A. (2015). Heritable symbiosis: The advantages and perils of an evolutionary rabbit hole. Proceedings of the National Academy of Sciences of the United States of America, 112, 10169–10176. 10.1073/pnas.1421388112 25713367 PMC4547261

[ece37900-bib-0005] Berghoff, S. M., Wurst, E., Ebermann, E., Sendova‐Franks, A. B., Rettenmeyer, C. W., & Franks, N. R. (2009). Symbionts of societies that fission: Mites as guests or parasites of army ants. Ecological Entomology, 34, 684–695. 10.1111/j.1365-2311.2009.01125.x

[ece37900-bib-0006] Bibian, A. J., Rudgers, J. A., & Miller, T. E. X. (2016). The role of host demographic storage in the ecological dynamics of heritable symbionts. The American Naturalist, 188, 446–459. 10.1086/687965 27622878

[ece37900-bib-0076] Bohn, H., Nehring, V., Rodríguez, J. G., & Klass, K.‐D. (2021). Revision of the genus Attaphila (Blattodea: Blaberoidea), myrmecophiles living in the mushroom gardens of leaf‐cutting ants. Arthropod Systematics & Phylogeny, (79), 205–280. 10.3897/asp.79.e67569

[ece37900-bib-0007] Bolivar, I. (1901). Un nuevo orthóptero mirmecófilo *Attaphila bergi*. Comunicaciones del Museo Nacional de Buenos Aires. Comunicaciones Del Museo Nacional De Buenos Aires, 1, 331–336.

[ece37900-bib-0008] Bonner, J. T. (2016). Life cycles: Reflections of an evolutionary biologist. Princeton University Press.

[ece37900-bib-0009] Brossut, R. (1976). Étude morphologique de la Blatte myrmécophile *Attaphila fungicola* Wheeler. Insectes Sociaux, 23, 167–174. 10.1007/BF02223849

[ece37900-bib-0010] Brown, J. K. (Ed.) (2016). Vector‐mediated transmission of plant pathogens. The American Phytopathological Society.

[ece37900-bib-0011] Bull, J. J. (1994). Virulence. Evolution, 48, 1423–1437. 10.1111/j.1558-5646.1994.tb02185.x 28568406

[ece37900-bib-0012] Bull, J. J. (2006). Optimality models of phage life history and parallels in disease evolution. Journal of Theoretical Biology, 241, 928–938. 10.1016/j.jtbi.2006.01.027 16616205

[ece37900-bib-0013] Bull, J. J., Molineux, I. J., & Rice, W. R. (1991). Selection of benevolence in a host‐parasite system. Evolution, 45, 875. 10.2307/2409695 28564051

[ece37900-bib-0077] Cahan, S., & Julian, G. E. (1999). Fitness consequences of cooperative colony founding in the desert leaf‐cutter ant Acromyrmex versicolor. Behavioral Ecology, 10(5), 585–591. 10.1093/beheco/10.5.585

[ece37900-bib-0014] Combes, C. (2001). Parasitism: The ecology and evolution of intimate interactions. University of Chicago Press.

[ece37900-bib-0015] Cremer, S., Pull, C. D., & Fürst, M. A. (2018). Social immunity: Emergence and evolution of colony‐level disease protection. Annual Review of Entomology, 63, 105–123. 10.1146/annurev-ento-020117-043110 28945976

[ece37900-bib-0016] Cremer, S., & Sixt, M. (2009). Analogies in the evolution of individual and social immunity. Philosophical Transactions of the Royal Society B: Biological Sciences, 364, 129–142. 10.1098/rstb.2008.0166 PMC2666697 18926974

[ece37900-bib-0017] Cronin, A. L., Molet, M., Doums, C., Monnin, T., & Peeters, C. (2013). Recurrent evolution of dependent colony foundation across eusocial insects. Annual Review of Entomology, 58, 37–55. 10.1146/annurev-ento-120811-153643 22934981

[ece37900-bib-0018] Day, T. (2003). Virulence evolution and the timing of disease life‐history events. Trends in Ecology & Evolution, 18, 113–118. 10.1016/S0169-5347(02)00049-6

[ece37900-bib-0019] Denny, A. J., Franks, N. R., Powell, S., & Edwards, K. J. (2004). Exceptionally high levels of multiple mating in an army ant. Naturwissenschaften, 91, 369–399. 10.1007/s00114-004-0546-4 15278220

[ece37900-bib-0020] Di Prisco, G., Pennacchio, F., Caprio, E., Boncristiani, H. F., Evans, J. D., & Chen, Y. (2011). Varroa destructor is an effective vector of Israeli acute paralysis virus in the honeybee, *Apis mellifera* . Journal of General Virology, 92, 151–155. 10.1099/vir.0.023853-0 20926637

[ece37900-bib-0021] Djernæs, M., Kotyková Varadínová, Z., Kotyk, M., Eulitz, U., & Klass, K.‐D. (2020). Phylogeny and life history evolution of Blaberoidea (Blattodea). Arthropod Systematics & Phylogeny, 78(1), 29–67. 10.26049/ASP78-1-2020-03

[ece37900-bib-0022] Ebert, D. (2013). The epidemiology and evolution of symbionts with mixed‐mode transmission. Annual Review of Ecology, Evolution, and Systematics, 44, 623–643. 10.1146/annurev-ecolsys-032513-100555

[ece37900-bib-0023] Fernández‐Marín, H., & Wcislo, W. T. (2005). Production of minima workers by gynes of *Atta colombica* Guérin‐Ménéville (Hymenoptera: Formicidae: Attini) that lack a fungal pellet. Journal of the Kansas Entomological Society, 78, 290–292. 10.2317/0402.19.1

[ece37900-bib-0024] Forti, L., Protti de Andrade, A., Camargo, R., Caldato, N., & Moreira, A. (2017). Discovering the giant nest architecture of grass‐cutting ants, *Atta capiguara* (Hymenoptera, Formicidae). Insects, 8, 39. 10.3390/insects8020039 PMC5492053 28350352

[ece37900-bib-0025] Fries, I., & Camazine, S. (2001). Implications of horizontal and vertical pathogen transmission for honey bee epidemiology. Apidologie, 32, 199–214. 10.1051/apido:2001122

[ece37900-bib-0026] Gaume, L., McKey, D., & Terrin, S. (1998). Ant–plant–homopteran mutualism: How the third partner affects the interaction between a plant‐specialist ant and its myrmecophyte host. Proceedings of the Royal Society of London. Series B: Biological Sciences, 265, 569–575. 10.1098/rspb.1998.0332

[ece37900-bib-0027] Genkai‐Kato, M., & Yamamura, N. (1999). Evolution of mutualistic symbiosis without vertical transmission. Theoretical Population Biology, 55, 309–323. 10.1006/tpbi.1998.1407 10366555

[ece37900-bib-0028] Golan, K., Rubinowska, K., Kmieć, K., Kot, I., Górska‐Drabik, E., Łagowska, B., & Michałek, W. (2015). Impact of scale insect infestation on the content of photosynthetic pigments and chlorophyll fluorescence in two host plant species. Arthropod‐Plant Interactions, 9, 55–65. 10.1007/s11829-014-9339-7

[ece37900-bib-0029] Hartmann, A. C., Baird, A. H., Knowlton, N., & Huang, D. (2017). The paradox of environmental symbiont acquisition in obligate mutualisms. Current Biology, 27, 3711–3716. 10.1016/j.cub.2017.10.036 29153324

[ece37900-bib-0030] Hartmann, A. C., Marhaver, K. L., Klueter, A., Lovci, M. T., Closek, C. J., Diaz, E., Chamberland, V. F., Archer, F. I., Deheyn, D. D., Vermeij, M. J. A., & Medina, M. (2019). Acquisition of obligate mutualist symbionts during the larval stage is not beneficial for a coral host. Molecular Ecology, 28, 141–155. 10.1111/mec.14967 30506836

[ece37900-bib-0031] Heineman, R. H., Springman, R., & Bull, J. J. (2008). Optimal foraging by bacteriophages through host avoidance. The American Naturalist, 171, E149–157. 10.1086/528962 18254683

[ece37900-bib-0032] Helms, J. A. IV (2018). The flight ecology of ants (Hymenoptera: Formicidae). Myrmecol. News, 26, 19–30. 10.25849/myrmecol.news_026:019

[ece37900-bib-0033] Herre, E., Knowlton, N., Mueller, U., & Rehner, S. (1999). The evolution of mutualisms: Exploring the paths between conflict and cooperation. Trends in Ecology & Evolution, 14, 49–53. 10.1016/S0169-5347(98)01529-8 10234251

[ece37900-bib-0034] Hölldobler, B., & Wilson, E. O. (2009). The superorganism: The beauty, elegance, and strangeness of insect societies (1st ed.). W.W. Norton.

[ece37900-bib-0035] Hughes, D. P., Pierce, N. E., & Boomsma, J. J. (2008). Social insect symbionts: Evolution in homeostatic fortresses. Trends in Ecology & Evolution, 23, 672–677. 10.1016/j.tree.2008.07.011 18951653

[ece37900-bib-0036] Hughes, W. O. H., Thomsen, L., Eilenberg, J., & Boomsma, J. J. (2004). Diversity of entomopathogenic fungi near leaf‐cutting ant nests in a neotropical forest, with particular reference to *Metarhizium anisopliae* var. *anisopliae* . Journal of Invertebrate Pathology, 85, 46–53. 10.1016/j.jip.2003.12.005 14992860

[ece37900-bib-0037] Iritani, R., Visher, E., & Boots, M. (2019). The evolution of stage‐specific virulence: Differential selection of parasites in juveniles. Evolution Letters, 3, 162–172. 10.1002/evl3.105 31289690 PMC6591554

[ece37900-bib-0038] Kronauer, D. J. C., Schoning, C., d’Ettorre, P., & Boomsma, J. J. (2010). Colony fusion and worker reproduction after queen loss in army ants. Proceedings of the Royal Society B: Biological Sciences, 277, 755–763. 10.1098/rspb.2009.1591 PMC2842746 19889701

[ece37900-bib-0039] LeBrun, E. G., Abbott, J., & Gilbert, L. E. (2013). Imported crazy ant displaces imported fire ant, reduces and homogenizes grassland ant and arthropod assemblages. Biological Invasions, 15, 2429–2442. 10.1007/s10530-013-0463-6

[ece37900-bib-0040] Leggett, H. C., Buckling, A., Long, G. H., & Boots, M. (2013). Generalism and the evolution of parasite virulence. Trends in Ecology & Evolution, 28, 592–596. 10.1016/j.tree.2013.07.002 23968968

[ece37900-bib-0041] Lipsitch, M., Siller, S., & Nowak, M. A. (1996). The evolution of virulence in pathogens with vertical and horizontal transmission. Evolution; International Journal of Organic Evolution, 50, 1729–1741. 10.1111/j.1558-5646.1996.tb03560.x 28565576

[ece37900-bib-0042] Łukasik, P., Newton, J. A., Sanders, J. G., Hu, Y. I., Moreau, C. S., Kronauer, D. J. C., O'Donnell, S., Koga, R., & Russell, J. A. (2017). The structured diversity of specialized gut symbionts of the New World army ants. Molecular Ecology, 26, 3808–3825. 10.1111/mec.14140 28393425

[ece37900-bib-0043] Marti, H. E., Carlson, A. L., Brown, B. V., & Mueller, U. G. (2015). Foundress queen mortality and early colony growth of the leafcutter ant, *Atta texana* (Formicidae, Hymenoptera). Insectes Sociaux, 62, 357–363. 10.1007/s00040-015-0413-7

[ece37900-bib-0044] Martin, S. J., Funch, R. R., Hanson, P. R., & Yoo, E.‐H. (2018). A vast 4,000‐year‐old spatial pattern of termite mounds. Current Biology, 28, R1292–R1293. 10.1016/j.cub.2018.09.061 30458144

[ece37900-bib-0078] Mintzer, A. C. (1987). Primary polygyny in the ant Atta texana: number and weight of females and colony foundation success in the laboratory. Insectes Sociaux, 34, 108–117.

[ece37900-bib-0045] Moreira, A. A., Forti, L. C., da Silva Camargo, R., Nagamoto, N. S., Caldato, N., Castellani, M. A., & Ramos, V. M. (2019). Variation in nest morphology, queen oviposition rates, and fungal species present in incipient colonies of the leaf‐cutter ant *Atta sexdens* . Tropical Zoology, 32, 107–117. 10.1080/03946975.2019.1603622

[ece37900-bib-0046] Moser, J. C. (1964). Inquiline roach responds to trail‐marking substance of leaf‐cutting ants. Science, 143, 1048–1049. 10.1126/science.143.3610.1048 17733069

[ece37900-bib-0047] Moser, J. C. (1967a). Mating activities of *Atta texana* (Hymenoptera, formicidae). Insectes Sociaux, 14, 295–312.

[ece37900-bib-0048] Moser, J. C. (1967b). Trails of leafcutters. The Journal of the American Museum of Natural History, 76, 33–37.

[ece37900-bib-0049] Moser, J. C. (2006). Complete excavation and mapping of a Texas leafcutting ant nest. Annals of the Entomological Society of America, 99, 891–897. 10.1603/0013-8746(2006)99[891:CEAMOA]2.0.CO;2

[ece37900-bib-0050] Moser, J. C., & Blomquist, S. R. (2011). Phoretic arthropods of the Red Imported Fire Ant in central Louisiana. Annals of the Entomological Society of America, 104, 886–894. 10.1603/AN11010

[ece37900-bib-0051] Mueller, U. G., Ishak, H. D., Bruschi, S. M., Smith, C. C., Herman, J. J., Solomon, S. E., Mikheyev, A. S., Rabeling, C., Scott, J. J., Cooper, M., Rodrigues, A., Ortiz, A., Brandão, C. R. F., Lattke, J. E., Pagnocca, F. C., Rehner, S. A., Schultz, T. R., Vasconcelos, H. L., Adams, R. M. M., … Bacci, M. (2017). Biogeography of mutualistic fungi cultivated by leafcutter ants. Molecular Ecology, 26, 6921–6937. 10.1111/mec.14431 29134724

[ece37900-bib-0052] Mueller, U. G., Mikheyev, A. S., Solomon, S. E., & Cooper, M. (2011). Frontier mutualism: Coevolutionary patterns at the northern range limit of the leaf‐cutter ant‐fungus symbiosis. Proceedings of the Royal Society B: Biological Sciences, 278, 3050–3059. 10.1098/rspb.2011.0125 PMC3158939 21389026

[ece37900-bib-0053] Nehring, V., Dani, F. R., Calamai, L., Turillazzi, S., Bohn, H., Klass, K.‐D., & d'Ettorre, P. (2016). Chemical disguise of myrmecophilous cockroaches and its implications for understanding nestmate recognition mechanisms in leaf‐cutting ants. BMC Ecology, 16. 10.1186/s12898-016-0089-5 PMC4974750 27495227

[ece37900-bib-0054] Parmentier, T. (2020). Guests of social insects. In C. Starr (Ed.), Encyclopedia of social insects (pp. 1–15). Cham: Springer.

[ece37900-bib-0056] Phillips, Z. I. (2021). Emigrating together but not establishing together: A cockroach rides ants and leaves. The American Naturalist, 197, 138–145. 10.1086/711876 33417528

[ece37900-bib-0055] Phillips, Z. I., Reding, L., & Farrior, C. E. (2021). Data from: The early life of a leaf‐cutter ant colony constrains symbiont vertical transmission and favors horizontal transmission. Ecology and Evolution, Dryad Digital Repository. 10.5061/dryad.8sf7m0cnt PMC8427574 34522335

[ece37900-bib-0057] Phillips, Z. I., Zhang, M. M., & Mueller, U. G. (2017). Dispersal of *Attaphila fungicola*, a symbiotic cockroach of leaf‐cutter ants. Insectes Sociaux, 64, 277–284. 10.1007/s00040-016-0535-6

[ece37900-bib-0058] Pull, C. D., Hughes, W. O. H., & Brown, M. J. F. (2013). Tolerating an infection: An indirect benefit of co‐founding queen associations in the ant *Lasius niger* . Naturwissenschaften, 100, 1125–1136. 10.1007/s00114-013-1115-5 24233126

[ece37900-bib-0059] R Core Team (2020). R: A language and environment for statistical computing. Vienna, Austria: R Foundation for Statistical Computing. http://www.R‐project.org/

[ece37900-bib-0060] Rettenmeyer, C. W., Rettenmeyer, M. E., Joseph, J., & Berghoff, S. M. (2011). The largest animal association centered on one species: The army ant *Eciton burchellii* and its more than 300 associates. Insectes Sociaux, 58, 281–292. 10.1007/s00040-010-0128-8

[ece37900-bib-0061] Rodríguez, J. G., Montoya‐Lerma, J., & Calle, Z. (2013). First record of *Attaphila fungicola* (Blattaria: Polyphagidae) in *Atta cephalotes* nests (Hymenoptera: Myrmicinae) in Colombia. Boletin Cientifico. Centro de Museos. Museo De Historia Natural, 17, 219–225.

[ece37900-bib-0062] Rynkiewicz, E. C., Pedersen, A. B., & Fenton, A. (2015). An ecosystem approach to understanding and managing within‐host parasite community dynamics. Trends in Parasitology, 31, 212–221. 10.1016/j.pt.2015.02.005 25814004

[ece37900-bib-0063] Sánchez‐Peña, S. R. (2005). Essays on organismal aspects of the fungus‐growing ant symbiosis: Ecology, experimental symbiont switches and fitness of Atta, and a new theory on the origin of ant fungiculture. University of Texas at Austin.

[ece37900-bib-0064] ten Brink, H., & de Roos, A. M. (2017). A parent‐offspring trade‐off limits the evolution of an ontogenetic niche shift. The American Naturalist, 190, 45–60. 10.1086/692066 28617644

[ece37900-bib-0065] Therneau, T. (2015). coxme: Mixed effects cox models. R package version 2.2‐3.

[ece37900-bib-0066] Tschinkel, W. R. (1993). Sociometry and sociogenesis of colonies of the Fire Ant *Solenopsis invicta* during one annual cycle. Ecological Monographs, 63, 425–457. 10.2307/2937154

[ece37900-bib-0067] Tschinkel, W. (2011). Back to basics: Sociometry and sociogenesis of ant societies (Hymenoptera: Formicidae). Myrmecological News, 14, 49–54.

[ece37900-bib-0068] Vargo, E. L., & Porter, S. D. (1989). Colony reproduction by budding in the polygyne form of *Solenopsis invicta* (Hymenoptera: Formicidae). Annals of the Entomological Society of America, 82, 307–313. 10.1093/aesa/82.3.307

[ece37900-bib-0069] Waller, D. A., & Moser, J. C. (1990). Invertebrate enemies and nest associates of the leaf‐cutting ant *Atta texana* (Buckley) (Formicidae, Attini). In R. K. Vander Meer, K. Jaffe, & A. Cedeno (Eds.), Applied myrmecology: A world perspective, Westview studies in insect biology. (pp. 255–273). Westview Press.

[ece37900-bib-0070] Werner, E. E., & Gilliam, J. F. (1984). The ontogenetic niche and species interactions in size‐structured populations. Annual Review of Ecology and Systematics, 15, 393–425. 10.1146/annurev.es.15.110184.002141

[ece37900-bib-0071] Wheeler, W. M. (1900). A new myrmecophile from the mushroom gardens of the Texan leaf‐cutting ant. The American Naturalist, 34, 851–862. 10.1086/277806

[ece37900-bib-0072] Wheeler, W. M. (1910). Ants: Their structure, development and behavior. Colombia University Press.

[ece37900-bib-0073] Wilson, E. O. (1985). The sociogenesis of insect colonies. Science, 228, 1489–1495. 10.1126/science.228.4707.1489 17831241

[ece37900-bib-0074] Woodard, S. H., Bloch, G., Band, M. R., & Robinson, G. E. (2013). Social regulation of maternal traits in nest‐founding bumble bee (*Bombus terrestris*) queens. Journal of Experimental Biology, 216, 3474–3482. 10.1242/jeb.087403 PMC4074288 23966589

[ece37900-bib-0075] Yang, A. S. (2007). Thinking outside the embryo: The superorganism as a model for EvoDevo studies. Biological Theory, 2, 398–408. 10.1162/biot.2007.2.4.398

